# Integrative Korean medicine treatment without surgery for the management of subacute radiating pain attributed to vertebral artery loop formation: A case report and literature review

**DOI:** 10.1097/MD.0000000000039483

**Published:** 2025-02-28

**Authors:** Jung Min Yun, Dong Hyun Go, Sook-Hyun Lee

**Affiliations:** a Department of Korean Medicine Rehabilitation, Jaseng Hospital of Korean Medicine, Gangnam-gu, Seoul, Republic of Korea; b Department of Public Health Sciences, Graduate School of Public Health, Seoul National University, Seoul, Republic of Korea; c Jaseng Spine and Joint Research Institute, Jaseng Medical Foundation, Gangnam-gu, Seoul, Republic of Korea.

**Keywords:** case report, cervical radiculopathy, EQ-5D index, integrative Korean medicine treatment, numerical rating scale, subacute, vertebral artery loop formation

## Abstract

**Rationale:**

Vertebral artery loop formation (VALF) compresses the adjacent cervical nerve root, resulting in cervical radiculopathy at the affected level. Neurosurgical interventions for anatomical separation are typically employed to relieve the symptoms, regardless of their duration. We herein report the management of the subacute symptoms of a rare case of cervical radiculopathy attributed to VALF using integrative Korean medicine (iKM) treatment without surgery.

**Patient concerns:**

A 54-year-old male patient presenting with a chief complaint of headache radiating to the posterior cervical and right scapular regions was treated with a 4-day program of inpatient care.

**Diagnoses:**

Based on the findings of the cervical spine magnetic resonance imaging, the patient was diagnosed with VALF on the right side at the C3/C4 level.

**Interventions:**

He underwent iKM treatment comprising acupuncture, pharmacopuncture, Chuna therapy, and herbal medicine.

**Outcomes:**

Significant improvement was noted in the patient’s condition (neck pain Numeric Rating Scale scores of 5, 4, and 1; Neck Disability Index scores of 35.56, 20, and 4; and EuroQol 5-dimension index scores of 0.754, 0.787, and 0.862 at admission, discharge, and the 2-month follow-up, respectively; Patient Global Impression of Change scores of 3 and 1 at discharge and the 2-month follow-up, respectively).

**Lessons:**

This case report suggests that iKM treatment may be effective for subacute VALF symptoms without requiring surgery.

## 1. Introduction

The vertebral artery (VA), which is a part of the vertebrobasilar vascular system, lies close to the cervical nerve root and ascends through the transverse foramen of the cervical vertebrae.^[1]^ VA loop formation (VALF) is a rare anatomical variant of the VA, which leads to compression of the adjacent cervical nerve root, resulting in symptoms associated with the level of cervical nerve root compression.^[2]^

Cervical radiculopathy is mainly associated with spine-related pathophysiology, such as disc herniation or spondylotic changes, resulting in compression of the cervical nerve root, or other pathologies, such as ectopic impulses in nerve conduction or inflammation, which are generally characterized by pain and tingling sensation or numbness.^[3,4]^ The incidence of VALF is relatively low (0.6–7.51%). Nevertheless, VALF adjacent to the cervical nerve root compresses the nerve root, similar to the nerve root compression by a herniated disc, resulting in cervical radiculopathy at the level of the affected cervical nerve root.^[5,6]^

Although case reports have reported a wide range of treatment modalities, including surgical^[7–11]^ and nonsurgical methods^[12,13]^ for managing conditions manifesting cervical radiculopathy attributed to VALF, owing to the small number of cases and heterogeneity in these reports, none of these treatment modalities have been firmly established in the literature.^[9,13,14]^

We herein report the management of the subacute symptoms attributed to VALF in a 54-year-old male patient using a 4-day integrative Korean medicine (iKM) program, and provide a literature review on the treatment modalities for the management of radiating pain attributed to VALF.

## 2. Case presentation

### 2.1. Patient history

A 54-year-old man presented with chief complaints of headache in the occipital area, pain in the posterior cervical and right scapular regions, and intermittent symptoms of numbness affecting the upper and lower arms. The onset of symptoms occurred after the patient received a massage at a public bathhouse in September 2022; he received inpatient care for 4 days in October 2022. He had no prior or existing illness that could have contributed to the radiating neck pain. No remarkable findings were noted in his family history or history of present illness.

### 2.2. Clinical findings

When the patient first visited the hospital on October 4, 2022, no remarkable findings were noted in terms of the range of motion (ROM) of the cervical spine. The results of bilateral examination with the Spurling test were negative; however, the patient reported aggravating headache around the posterior cervical region during flexion of the cervical spine, with neck pain up to the occipital area. Regardless of the arm, wrist, and finger movement, the muscle strength assessment of the right arm showed Grade 5. Reflex function examination and sensory testing of the right arm revealed no abnormal findings or sensory loss, respectively.

**Figure 1.**
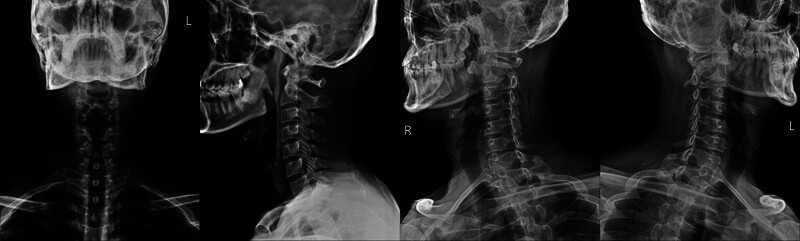
X-ray image scan of the cervical spine. C-spine series (anterior to posterior, lateral, oblique) was taken, and no remarkable findings were found other than straightening of cervical spine curvature.

**Figure 2.**
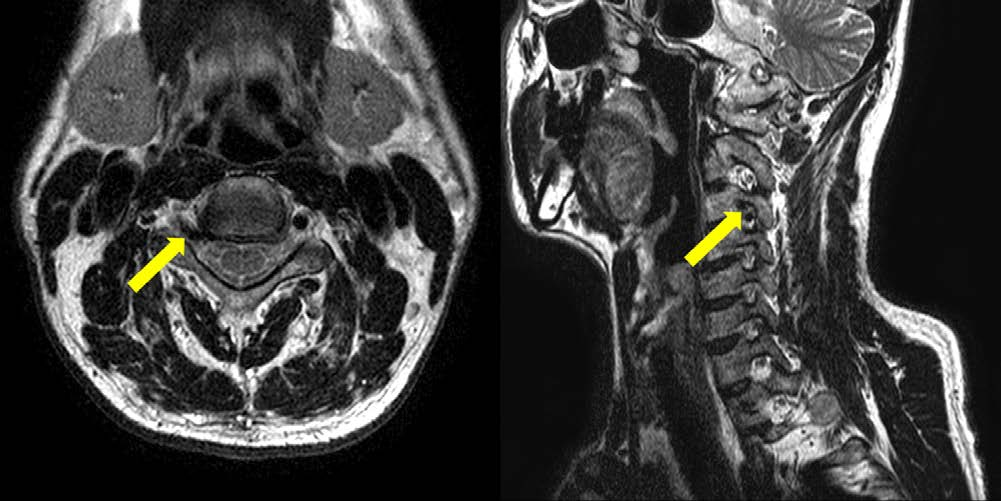
T2-weighted magnetic resonance imaging (MRI) scan of the cervical spine. Axial section at C3/4 (left) and oblique section (right). The axial section shows compression of the nerve root (yellow arrow) by the vertebral artery loop around the foraminal zone at the right C3/4 level. The oblique section shows a vertebral artery flow void instead of a nerve root at the right C3/4 level (yellow arrow).

Since the physical examination revealed no abnormal findings, cervical spine (C-spine) imaging was performed on the same day. First, X-ray imaging was performed to generally screen the pathological condition of C-spine (Fig. 1). The radiographs demonstrated no findings indicative of radiating pain; thus, C-spine magnetic resonance imaging (MRI) was performed for further examination. MRI revealed right VALF at the C3/4 level, a bulging disc at the C4/5, C5/6, and C6/7 levels, and mild subarticular disc protrusion at the T1/2 level (Fig. 2). As the signs of disc herniation were not pronounced at the C4/5, C5/6, and C6/7 levels, the symptoms of headache and radiating pain in the posterior cervical region of the patient were considered to be attributed to the right VALF at the C3/4 level. Additionally, the intermittent symptoms of numbness in the right upper and lower arms were considered to be associated with the pathophysiology at the T1/2 disc level.

### 2.3. Therapeutic intervention

The therapeutic interventions included acupuncture, pharmacopuncture, Chuna therapy, herbal medicine, cupping therapy, and physical therapy.

Acupuncture was performed 6 times during the treatment period. Sterile stainless steel filiform needles with diameters of 0.25 × 30 mm and 0.30 × 60 mm (Dongbang Acupuncture Inc., Boryeong, Korea) were used. Ten needles were inserted and maintained at a depth of approximately 30 mm for 15 minutes. Acupuncture was performed twice daily, in the morning and afternoon, excluding the days of admission and discharge. Electrical stimulation of the needles and transcutaneous infrared therapy were performed simultaneously at 2 Hz during needle retention. The acupoints were GB20 (Feng chi) and SI04 (Wan gu) in the posterior cervical region, SI15 (Jian zhong yu), GB21 (Jian jiang), and SI11 (Tian zong) in the shoulder and back region, and Ashi-points in the peripheral region.

Six pharmacopuncture sessions were conducted during the treatment. Pharmacopuncture was administered along with acupuncture twice daily in the morning and afternoon, excluding the days of admission and discharge. Shinbaro pharmacopuncture solution (Jaseng Pharmacopuncture Research Institute, Namyangju, S. Korea) prepared at the Jaseng Herbal Dispensary, an extramural facility, was used, which was administered using a 10-mL disposable syringe (Kovax-Syringe; Korea Vaccine, Ansan, S. Korea) and a 38-mm needle (30 G × 38 mm [1-1/2”]; Jung Rim, Jincheon, S. Korea). For the acupoints, the nerve root around the right facet joint at the C3/4 level were selected, and an 8-mL pharmacopuncture solution was injected into the muscle at a depth of approximately 3 cm perpendicular to the skin. The treatment area was disinfected with a 10% povidone–iodine solution before the procedure to prevent infection.

Chuna therapy was administered on the neck region, and cervical spine and upper thoracic spine adjustment techniques were applied according to the patient’s condition; the status of correction in the alignment was examined through reevaluation. Chuna therapy was administered once daily for 10 minutes before each acupuncture session. Techniques were applied to correct any misalignment in the cervical spine and relieve the tension in the adjacent soft tissues.

Herbal medicines were prescribed on admission and at discharge. The types of herbal medicine used during the treatment period were Geoseub-hwalhyeol-jitong-tang, Singyeongbaro-hwan, Hyungbangjihwang-tang, and Jasenglyeog-go; the herbal ingredients and doses are summarized in Table S1, Supplemental Digital Content, http://links.lww.com/MD/N464. Geoseub-hwalhyeol-jitong-tang and Singyeongbaro-hwan were prescribed at admission for pain relief and to improve blood flow. For Geoseub-hwalhyeol-jitong-tang, 2 packs of the preparation were divided into 3 portions to be taken 3 times daily, 30 minutes after meals; whereas, for Singyeongbaro-hwan, 3 bags of the herbal medicine were divided into 3 portions to be taken 3 times daily, 30 minutes after meals. The same prescription at admission was administered until the second day after discharge. From the 3rd day after discharge, Hyungbangjihwang-tang, which is used with Soyangin and Jasenglyeog-go for improving blood flow, was prescribed considering the patient’s constitution according to the Sasang constitutional medicine classification. A total of 40 packs of Hyungbangjihwang-tang were provided for 30 days; the patient was instructed to take the preparation twice daily in the morning and evening, 30 minutes after meals. For Jasenglyeog-go, 2 bags were taken daily, 30 minutes after meals in the morning and evening, for 30 days. During the treatment period, no medications other than the prescribed herbal medicines, including conventional medicines, were taken.

For cupping therapy, sterile cups (Dongbang cupping cup No. 2; DongBang Medical, Seongnam, S. Korea) were used at 2 sites of tenderness along the applicable neural pathway in the neck and scapular regions. Cupping therapy was administered twice daily for 10 minutes with acupuncture. Physical therapy was administered for 3 days, excluding the day of admission. Ultrasound interferential current therapy and laser therapy were performed once daily for 30 minutes in the posterior cervical region.

### 2.4. Follow-up and outcomes

**Figure 3.**
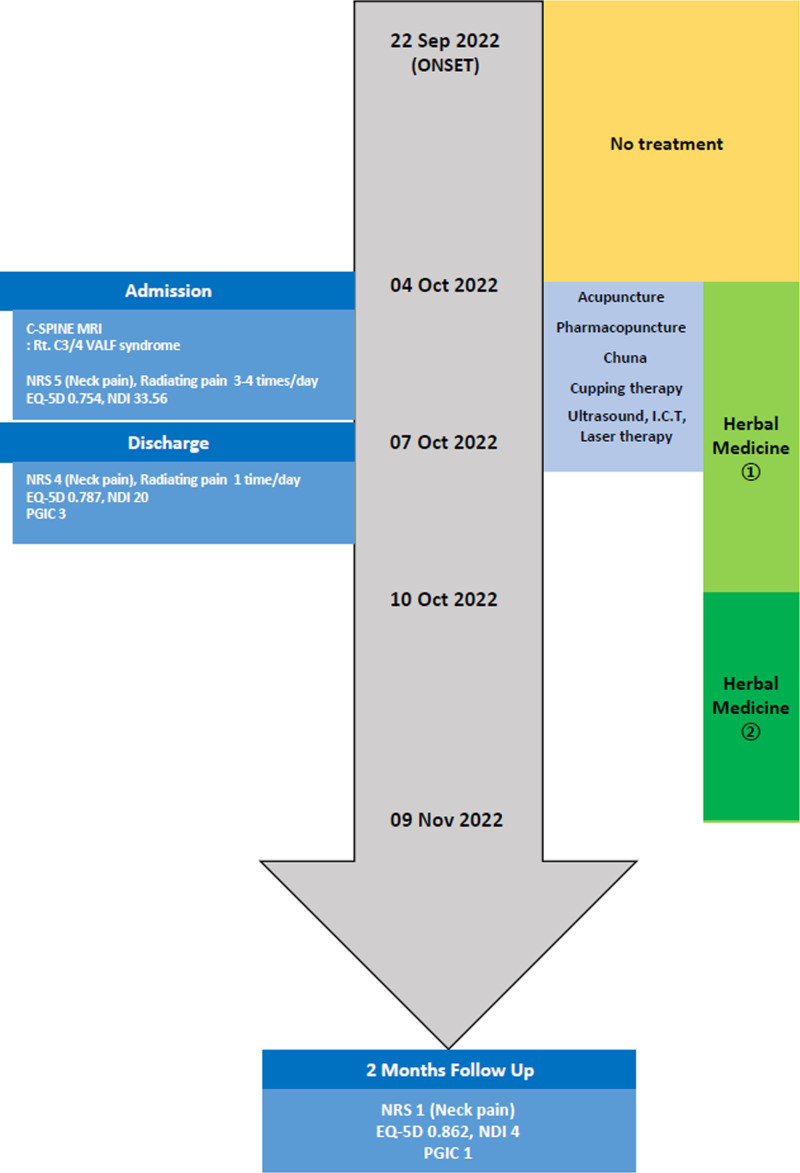
Timeline of integrative Korean medicine treatment and the changes in the outcome. EQ-5D index = EuroQol five-dimension index, NDI = Neck Disability Index, NRS = numerical rating scale, PGIC = Patient Global Impression of Change.

The outcomes measured at different time points during the treatment period are summarized in Table 1. The following 4 measures were used to evaluate the patient’s symptoms: numerical rating scale (NRS), EuroQol five-dimension index, Neck Disability Index (NDI), and Patient Global Impression of Change (PGIC). On admission (October 4), the NRS score for neck pain was 5, and the ROM evaluations showed normal findings on usual days; however, the patient reported pain in the right scapular region, neck pain, and headache when slanting his neck forward for a long duration. The average frequency of occurrence of these symptoms was 3 to 4 times daily. The patient was afraid to move his neck during the occurrence of neck pain owing to the fear of onset of radiating pain. The patient also complained of neck discomfort when he tried to look at an object on the floor, and the symptoms affected his concentration, resulting in a score of 0.754 on the EuroQol five-dimension index and 35.56 on the NDI. From October 5 to October 6, the frequency of radiating pain, including pain in the right scapular region, neck pain, and headache, was reduced to once daily; however, the patient expressed that the severity of the pain was the same as that before pain occurrence, with an NRS score of 5. To increase the ROM and facilitate the patient’s return to work, the patient was discharged on the 4th day after admission. At discharge, the NRS, EQ-5D, NDI, and PGIC scores were 4, 0.787, 20, and 3 (minimal improvement), respectively, indicating slight improvement in symptoms on all 4 scales. The patient reported radiating pain occurring once at discharge; however, he expressed reduction in the pain intensity over time, improved concentration, and reduction in his fear of developing radiating pain due to neck movement. After discharge, herbal medicine treatment was continued for the symptoms of radiating neck pain, and the patient did not visit other hospitals or receive additional medical treatments. The patient did not mention any other adverse events, such as dizziness or vomiting, during the treatment period. In the 2-month follow-up assessment, the NRS, EQ-5D, NDI, and PGIC scores were 1, 0.862, 4, and 1 (very much improved), respectively, indicating considerable improvement in pain, functional disability, and quality of life. The timeline of the treatment provided to the patient and changes in the symptom-related outcomes are presented in Figure 3 and Table 1.

**Table 1:** Symptom evaluation results on the 4 scales considered in this case report.

	Admission	1 day	2 days	Discharge	2 months
NRS	5	5	5	4	1
EQ-5D index	0.754	–	–	0.787	0.862
NDI	35.56	–	–	20	4
PGIC	–	–	–	3	1

EQ-5D index = EuroQol five-dimension index, NDI = Neck Disability Index, NRS = numerical rating scale, PGIC = Patient Global Impression of Change.

## 3. Discussion

**Figure 4.**
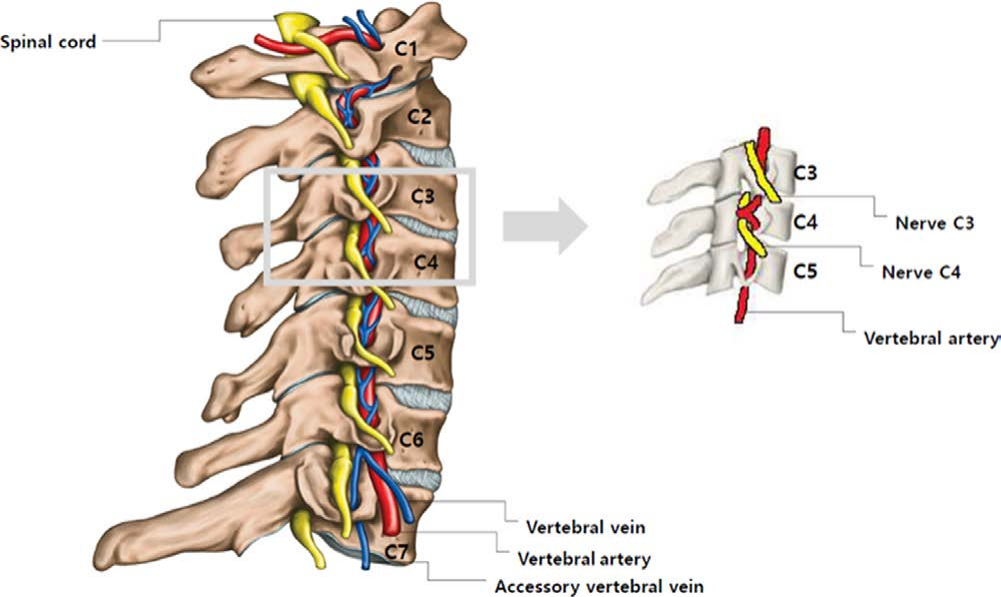
A schematic illustration of the right C3 to C4 vertebral artery loop compressing the nerve root.

Although patients with cervical radiculopathy attributed to VALF exhibit pain similar to that in those with cervical radiculopathy of different pathophysiologies, VALF-induced cervical radiculopathy is a rare clinical condition. The VAs are segmented with reference to the cranium, and the V1 to V3 segments are located extracranially, whereas the V4 segment is situated intracranially. The V2 segment, located between the transverse foramina of C6 to C2, accounts for most of the cases (90.5%) of symptomatic VALF.^[5,9,13]^ Herein, we present a case of cervical radiculopathy caused by compression of the right C4 nerve root (V2 segment) by a VALF (Fig. 4).

Clinically, in the case of radiating pain related to the cervical spine, it is easy to judge that it is a symptom caused by degenerative changes in the spine such as herinated disc or spinal stenosis. In this case report, considering only the patient’s age and the symptoms of radiating pain he complained of, it was easy to determine that the disease was related to degenerative changes in the cervical spine. However, when referring to physical examination and X-ray image scan, it was confirmed that degenerative changes in the cervical spine were unlikely to cause radiating pain, and an additional MRI was performed. On MRI, it was confirmed that radiating pain caused by VALF occurred rather than a pathological condition of the disc, and we treated the patient with the goal of treating the radiating pain symptoms caused by VALF.

Previous studies on hand acupuncture reported significantly reduced blood flow volume and velocity in the VAs of healthy individuals,^[15]^ and the stimulation provided by acupuncture triggers the release of opioid peptides, such as β-endorphins, enkephalins, and dynorphins, in the central nervous system, thus inducing the analgesic effect.^[16,17]^ Although acupuncture was not administered to the hand area for treatment in this study, it played a major role in improving the hemodynamics associated with discomfort in the head and neck of the patient. Shinbaro pharmacopuncture reportedly demonstrates anti-inflammatory activity and reduces pain by increasing the pain threshold.^[18]^ In addition, in this case, pharmacopuncture also includes the meaning of hydrodissection. Chuna therapy can serve as a safe and effective treatment method for various musculoskeletal disorders,^[19]^ especially acute or subacute neck pain.^[20]^ Ultimately, the pathological cause of this case was compression of the nerve root due to VALF, so we attempted to correct the misalignment of the cervical spine and physically separate VA from the nerve root. The main goal of using herbal medicine was to relieve the patient’s radiating pain and improve blood flow. Geoseub-hwalhyeol-jitong-tang may provide anti-inflammatory effects and pain relief and improve blood flow.^[21]^ Singyeongbaro-hwan is a prescription used by our clinic to relieve radiating pain caused by nerve stimulation. Hyungbangjihwang-tang, a prescription of Sasang constitutional medicine for Soyangin patients, reportedly inhibits lipopolysaccharide-induced inflammatory cytokine production; the herbal medicine prescription has various clinical applications, such as treatment of headache, fatigue, lower back pain, or indigestion.^[22,23]^ Jasenglyeog-go is a prescription based on Yukmijihwang-Tang with the addition of drugs to enhance anti-inflammatory effects and blood circulation. Yukmijihwang-tang is a famous herbal prescription used to relieve “Soyangin patients Yin-Deficiency” in iKM, and reportedly has antioxidant effects.^[24,25]^Cupping therapy and physical therapy have been applied as adjunctive therapies for their anti-inflammatory and analgesic effects and blood flow improvement.

A literature review was conducted on the treatment of radiating pain caused by VALF syndrome; the protocol was registered in the Open Science Framework (DOI: 10.17605/OSF.IO/D9UHK). The searches were conducted using the PubMed database; 22 articles were identified (Figure S1, Supplemental Digital Content, http://links.lww.com/MD/N463 and Tables S2, Supplemental Digital Content, http://links.lww.com/MD/N465 and S3, Supplemental Digital Content, http://links.lww.com/MD/N466). All the 22 articles were case reports. For the treatment of VALF syndrome, 11 patients underwent surgical intervention, 9 underwent surgical intervention after conservation therapy, and 4 underwent conservative therapy only. Surgical pledgets were used to prevent the VA from compressing the nerve root, and anatomical resection was used to enlarge the regional space around the VA. Considering the case reports describing conservative approaches for managing VALF-induced symptoms, medications were mainly used; other modalities included epidural injections and rehabilitation programs. However, specific descriptions of the duration of the treatment or details of the intervention are lacking in these reports, making the evaluation of their therapeutic effects challenging.

Through a literature review, we learned that the treatments for radiating pain caused by VALF are still controversial. In summary, surgical interventions were used to prevent the VA from physically compressing the nerve root. Conservation therapy, including medication, was used to relieve pain through biochemical methods, focusing on radiating pain caused by VALF. Regardless of whether physical or biochemical methods were used, treatments were needed to block specific parts of the entire process in which VALF compresses the nerve root and the patient felt pain. In other words, due to the nature of VALF syndrome, it was difficult to expect spontaneous improvement unless VA was normalized, especially within a short period of time.

What is important to note here is that the iKM we performed was performed from a comprehensive perspective, including physical and biochemical methods, and this treatment effectively relieved pain for 2 months. Pharmacoupuncture and Chuna therapy were used to physically prevent VA from compressing the nerve roots and correct alignment of the cervical spine, and physiochemical methods such as acupuncture and herbal medicine were used to relieve radiating pain. According to Table S3 , Supplemental Digital Content, http://links.lww.com/MD/N466, there were cases where pain was immediately alleviated using paramedian dural incision, hemilaminectomy, surgical pledgets, coiling technique, etc. However, in cases where other surgical interventions, medication, and epidural injection were used, there were few cases where pain relief was achieved within 2 months. iKM used in this case report may be more effective than other interventions in that it effectively reduced pain within 2 months.

There are some limitations to the treatment approach employed in this case report. First, the length of hospital stay for the patient was only 4 days; after discharge, the patient continued with only herbal medicine treatment and did not visit the hospital, which limited examining the trend over time for the 4 scales used for outcome assessment of the patient. In general, the effect of conservative treatment is not as immediate as in the case of surgical treatment. However, during the period of hospital stay, the frequency of radiating pain reduced from 3 to 4 times a day to once a day, and after discharge, the patient was able to return to his daily life without requiring additional treatment other than herbal medicine or a visit to the hospital. Second, the patient in this case report had symptoms of cervical radiculopathy for approximately 1 month. However, subacute cases have not been previously reported, and cases with a somewhat chronic course have been published.^[14,26,27]^ Hence, the findings of our case report cannot be generalized to predict the effects of iKM in patients with chronic conditions. Third, compared to surgical treatment, which reduces radiculopathy symptoms by physically separating the VA compressing the nerve root, pharmacopuncture and Chuna therapy of iKM treatment helped physically separate the VA compressing the nerve root. However, since MRI could not be taken again after treatment, it was difficult to assess whether physical separation had occurred accurately. Lastly, magnetic resonance angiography may be considered useful for achieving accurate imaging and observation of loop formation in the VA. However, the image of the VA forming a loop in the horizontal plane should be inferred with evidence of signal void tubular structures on MRI.^[28–30]^

Nevertheless, this case report and literature review provides a detailed description of the types of conservative interventions, methods, and duration of treatment for VALF-induced cervical radiculopathy; moreover, the assessment of outcomes indicated conservative treatment to be effective in nonchronic patients with VALF. Furthermore, a conservative approach may be considered as the initial mode of care before considering surgery in patients with VALF with moderate pain. We were unable to describe the adverse effects of surgical treatment as most studies did not describe the adverse effects. However, as surgical treatment is an invasive approach, the possibility of soft tissue injury arising from incisions in the neck area or complications attributed to the insertion of artificial objects cannot be ruled out. Moreover, patients may be hesitant to undergo reoperation if they have previously undergone several surgeries in the concerned area. Thus, the proposed iKM treatment may serve as a useful option to resolve these limitations.

This case report presents the management of a patient with cervical radiculopathy attributed to VALF using an iKM treatment program. During treatment, the patient demonstrated continuous improvement in radiating neck pain, function, and quality of life. Since the incidence of VALF is low, the efficacy of various treatment modalities is controversial, even for surgical approaches, and few high-quality studies have been reported to date. In the future, more data on nonsurgical treatments should be collected, supported by studies with high-quality evidence.

## Author contributions

**Conceptualization:** Jung Min Yun, Dong Hyun Go.

**Data curation:** Jung Min Yun.

**Formal analysis:** Jung Min Yun, Sook-Hyun Lee.

**Investigation:** Jung Min Yun, Dong Hyun Go.

**Supervision:** Sook-Hyun Lee.

**Writing – original draft:** Jung Min Yun.

**Writing – review & editing:** Sook-Hyun Lee.

## Supplementary Material


