# Supplementary material for: Integrative Korean medicine treatment without surgery for the management of subacute radiating pain attributed to vertebral artery loop formation: A case report and literature review
Source: Medicine (Baltimore). 2025 Feb 28;104(9):e39483. doi: 10.1097/MD.0000000000039483 (PMC11875575; doi:10.1097/MD.0000000000039483)
Supplement: Supplementary file 1 [file medi-104-e39483-s001.docx]

**Table S1**. Composition and amount of herbal medicine used in this case

| Herbal medicine  (Decoction, pill, paste) | Contents* |
| --- | --- |
| Geoseub-hwalhyeol-jitong-tang  (祛濕活血止痛湯) | 金銀花 7.5 g, 木通 7.5 g, 薏苡仁 7.5 g, 蒼朮 7.5 g, 桂枝 3.75 g, 桂枝 3.75 g, 當歸 3.75 g, 赤芍藥 3.75 g, 桃仁 3.75 g, 防風 3.75 g, 白芷 3.75 g, 生地黃 3.75 g, 烏藥 3.75 g, 牛膝 3.75 g, 威靈仙 3.75 g, 陳皮 3.75 g, 赤茯苓 3.75 g, 川芎 3.75 g, 龍膽 3.75 g, 萹蓄 3.75 g, 蘇木 1.875 g, 紅花 1.875 g |
| Singyeongbaro-hwan | 白茯苓 1.320 g, 人蔘 0.660 g, 牛膝 0.058 g, 阿膠 0.029 g, 狗脊 0.310 g, 白朮 0.310 g, 附子 0.086 g, 桂皮 0.054 g, 乾薑 0.032 g, 畢撥 0.032 g, 桂枝 0.021 g |
| Hyungbangjihwang-tang  (荊防地黃湯) | 熟地黃 7.5 g, 山茱萸 7.5 g, 白茯苓 7.5 g, 澤瀉 7.5 g, 車前子 3.75 g, 羌活 3.75 g, 獨活 3.75 g, 荊芥 3.75 g, 防風 3.75 g |
| Jasenglyeog-go | 生地黃 3.454 g, 鹿茸 0.332 g, 當歸 0.332 g, 山藥 0.332 g, 山茱萸 0.332 g, 白茯苓 0.166 g, 澤瀉 0.166 g, 牡丹皮 0.166 g, 沈香 0.018 g, 紫荷蕖 0.018 g, 蜂蜜 4.960 g |

*Dosage per pack
