# Supplementary figures and images for: Integrative Korean medicine treatment without surgery for the management of subacute radiating pain attributed to vertebral artery loop formation: A case report and literature review
Source: Medicine (Baltimore). 2025 Feb 28;104(9):e39483. doi: 10.1097/MD.0000000000039483 (PMC11875575; doi:10.1097/MD.0000000000039483)

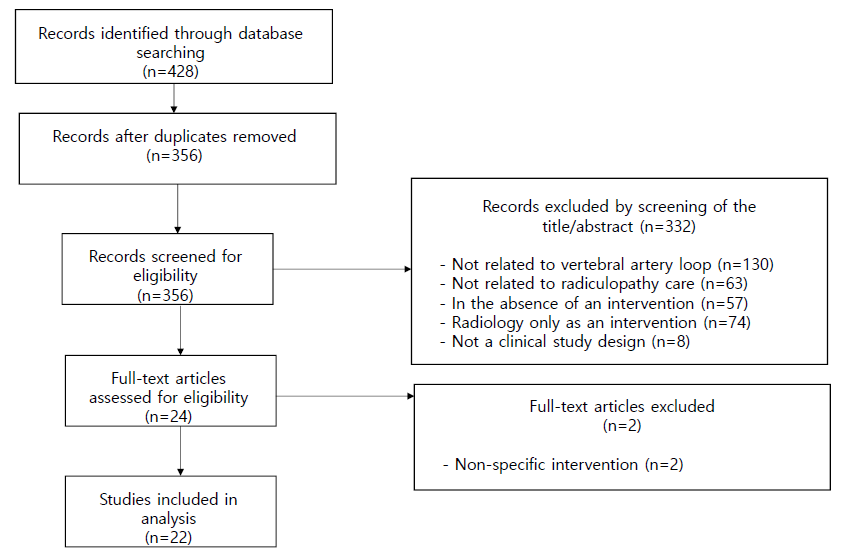


**Figure S1.** Flow chart for searching papers on PubMed.

Supplement: Supplementary file 2 [file medi-104-e39483-s002.docx]
