# Supplementary material for: Integrative Korean medicine treatment without surgery for the management of subacute radiating pain attributed to vertebral artery loop formation: A case report and literature review
Source: Medicine (Baltimore). 2025 Feb 28;104(9):e39483. doi: 10.1097/MD.0000000000039483 (PMC11875575; doi:10.1097/MD.0000000000039483)
Supplement: Supplementary file 3 [file medi-104-e39483-s003.docx]

**Table S2.** PubMed database electronic search strategy

| **Search number** | **Terms/Combinations** | **Results** |
| --- | --- | --- |
| #1 | (vertebral artery) AND (loop) | 205 |
| #2 | (vertebral artery) AND (radiculopathy) | 179 |
| #3 | (vertebral artery [MeSH Terms]) AND (radiculopathy [MeSH Terms]) | 44 |
| Strategy | #1 OR #2 OR #3 | 356 |

**Search date: 2024.01.29**
