# Supplementary material for: Integrative Korean medicine treatment without surgery for the management of subacute radiating pain attributed to vertebral artery loop formation: A case report and literature review
Source: Medicine (Baltimore). 2025 Feb 28;104(9):e39483. doi: 10.1097/MD.0000000000039483 (PMC11875575; doi:10.1097/MD.0000000000039483)
Supplement: Supplementary file 4 [file medi-104-e39483-s004.docx]

**Table S3.** Characteristics of the studies included in the literature review

| **Study** | **Design** | **Intervention** | | **Symptoms**  **(Side/Nerve root level/specified)** | **Age/**  **Sex** | **Outcome** |
| --- | --- | --- | --- | --- | --- | --- |
| **Using only surgical interventions** | | | | | | |
| Wood et al. (2021) | Case report^†^ (2 cases) | Surgical pledgets,  Microvascular decompression | | Case 1: Left/C6/Left arm and neck pain | Case 1: 35/M | Case 1: Asymp. within 3 months |
| Rigal et al. (2022) | Case report^†^ | Lateral mass resection | | Left/C6/Left C6 dermatome and neck pain | 51/F | Asymp. in 3 months |
| Goel et al. (2021) | Case report | Transarticular screw fixation | | Both/C2/Difficulty in walking and tingling paresthesias in all 4 limbs | 50/F | Asymp. in 22 months |
| Venteicher et al. (2019) | Case report^†^ | Surgical pledgets,  Microvascular decompression | | Right/C5/Right arm and shoulder pain, right deltoid weakness | 72/F | Asymp. in 1 year |
| Hage et al. (2012) | Case report | Drilling,  Microvascular decompression | | Right/C7/Right hand pain, numbness and tingling, neck pain | 27/F | Some reduction in 8 months(Residual right fourth and fifth digits paresthesia) |
| Duthel et al. (1994) | Case report | Surgical pledgets,  Microvascular decompression | | Left/C6/Left shoulder pain and second, third fingers acroparesthesia and C6,7 dermatome pain | 37/M | Asymp. in 3 months |
| Sakaida et al. (2001) | Case report | Vascular reconstruction | | Left/C5/Left shoulder pain and arm pain with dizziness | 62/M | Asymp. in 2 years |
| Omura et al. (2020) | Case report | Surgical material prosthesis,  Microvascular decompression, | | Left/C1/ Neck pain while turning his neck with dizziness, ataxia, and spasticity | 72/M | Asymp. in 10 years |
| Trimble et al. (2011) | Case report^††^ | Surgical pledgets,  Microvascular decompression | | Left/C2/Left suboccipital and posterior neck pain | 40/M | Some reduction in 18 months (residual neck pain) |
| Yamada et al. (2018) | Case report | Paramedian dural incision, Microvascular decompression, | | Left/C1/Left neck pain induced by neck flexion, occipitalgia, and sensory abnormalities in the left arm | 30/F | Some reduction in the post-operative period |
| Wang et al. (2017) | Case report^†^  (2 cases) | Microvascular decompression | | Case 2: Left/C4/Left neck and occiput pain | Case 2:  49/F | Some reduction in 6 weeks |
| **Using surgical interventions after conservative treatment** | | | | | | |
| Chibbaro et al. (2012) | Case report^†^ | NS | Microvascular decompression | Left/C6/Left radiculopathy | 50/F | Asymp. in 3 days |
| Farshad et al. (2021) | Case report | Physiotherapy,  Acupuncture | ARDF | Right/C6/Right arm and thumb pain | 76/F | Asymp. in 1 year |
| Wood et al. (2021) | Case report^†^ (2 cases) | Botox injections,  Nerve blocks | Surgical pledgets,  Microvascular decompression | Case 2: Left/C4,5/Left shoulder pain and middle three fingers pain, paresthesia and weakness | Case 2: 48/F | Case 2: Some reduction in 6 months (residual shoulder pain) |
| Detwiler et al. (1998) | Case report | Epidural injections,  Medications(Anti-inflammatory),  Trigger-point injections, Physiotherapy | Hemilaminectomy,  Facetectomy,  Microvascular decompression | Right/C4/Right shoulder and neck pain | 70/M | Asymp. in the post-operative period |
| Ju et al. (2017) | Case report | NS | Sling technique,  Microvascular decompression | Left/C7/Left arm pain and numbness | 52/F | Asymp. in 10 days |
| Khansuheb et al. (2020) | Case report^†^ | Epidural injections,  Trigger point injections | Coiling technique | Left/C6,7/Left neck pain and middle three fingers pain, numbness, and tingling | 62/F | Some reduction in the post-operative period |
| Korinth et al. (2006) | Case report | Medications (Anti-inflammatory),  Physiotherapy | Surgical pledgets,  Microvascular decompression | Right/C5/Right neck, shoulder, and arm pain | 68/F | Asymp. in the post-operative period |
| Rho et al. (2017) | Case report | Epidural injections,  Medications(Anti-inflammatory),  Trigger point injections,  Physiotherapy | Hemilaminectomy,  Facetectomy,  Transarticular screw fixation | Left/C6/Left shoulder and neck pain | 51/M | Asymp. in 3 days |
| Wang et al. (2017) | Case report^†^ (2 cases) | NS | Surgical pledgets,  Microvascular decompression | Case 1: Left/C6/Left C6 dermatome pain, paresthesia | Case 1:  51/F | Asymp. in 4 months |
| **Using only conservative interventions** | | | | | | |
| Tack et al. (2021) | Case report^†^ | Medications (Pregabalin),  Rehabilitation program | | Left/C4/Left arm and neck pain | 70/M | Asymp. |
| Horgan et al. (1998) | Case report | Medications (NSAIDs) | | Right/C4,5/Right neck and shoulder pain, and headaches | 66/F | Non-progressive in 4 years |
| Croci et al. (2020) | Case report | Epidural injections | | Right/C7/Right arm, shoulder and neck pain | 37/M | Asymp. in 8 months |
| Naldemir et al. (2020) | Case report^†^ | Medications (NSAIDs) | | Right/C4/Right shoulder and neck pain | 19/F | NS |

Asymp: The patient was asymptomatic. NS: Not specified. ARDF: Anterior artery release, distraction, and fusion, NSAIDs: non-steroidal anti-inflammatory drugs.

†: Cases reporting patients who have already undergone other surgical interventions.

†† : This case report describes a patient who previously underwent radiofrequency ablation.

Wood et al. (2021) and Wang et al. (2017) each presented two cases. Case 1 and Case 2 of Wood et al. (2021) and Wang et al. (2017) were written separately in a table.
